# Kinase inhibition rewires the HLA-I immunopeptidome in chronic myeloid leukemia

**DOI:** 10.1016/j.isci.2026.115944

**Published:** 2026-04-30

**Authors:** Veronica Venafra, Maria Wahle, Giorgia Massacci, Valeria Bica, Patrizia Chiusolo, Dimitrios Mougiakakos, Martin Boettcher, Thomas Fischer, Livia Perfetto, Matthias Mann, Francesca Sacco

**Affiliations:** 1 Department of Biology and Biotechnologies “Charles Darwin”, University of Rome La Sapienza, Piazzale Aldo Moro 5, 00185 Rome, Italy; 2 Department Proteomics and Signal Transduction, Max Planck Institute of Biochemistry, Am Klopferspitz 18, 82152 Planegg, Germany; 3 Department of Biology, University of Rome Tor Vergata, Via della Ricerca Scientifica 1, 00133 Rome, Italy; 4 Sezione di Ematologia, Dipartimento di Scienze Radiologiche ed Ematologiche, Università Cattolica del Sacro Cuore, Largo Agostino Gemelli 8, 00168 Rome, Italy; 5 Health Campus for Inflammation, Immunity and Infection (GCI3), Otto-von-Guericke University of Magdeburg, Universitätspl. 2, 39106 Magdeburg, Germany; 6 Department of Hematology, Oncology and Cell Therapy, Otto-von-Guericke University of Magdeburg, Universitätspl. 2, 39106 Magdeburg, Germany; 7 Telethon Institute of Genetics and Medicine (TIGEM), Via dei Campi Flegrei 34, 00141 Pozzuoli, Naples, Italy

**Keywords:** oncology, immunology, cell biology

## Abstract

Immunotherapy offers promising opportunities to improve immune-mediated control of chronic myeloid leukemia (CML), but its success depends on identifying antigens uniquely associated with CML. Protein kinases regulate signaling and protein turnover, influencing which peptides are presented on HLA molecules. Although earlier studies suggested that kinase inhibition can alter immune recognition of cancer cells, the underlying mechanisms remain unclear. In this study, we investigated whether targeting key kinases could reshape the CML immunopeptidome. The pharmacological inhibition of SFK, JNK, and BCR-ABL caused broad remodeling of antigen presentation, affecting the display of 4,000 HLA-I ligands. By integrating immunopeptidomics, phospho-immunopeptidomics, and proteomics, we identified three complementary mechanisms regulating antigen display. Comparing benign hematologic tissues with CML samples revealed about 90 CML-specific peptides that became more prominent after SFK or JNK inhibition. Overall, this work provides a quantitative, multi-omic framework to rationally combine kinase inhibitors with immunotherapy to boost antigen visibility and antileukemic immunity.

## Highlights

• Kinase signaling reshapes the HLA-I immunopeptidome in CML
• SFK and JNK inhibition increase presentation of immunogenic TAAs
• Phosphorylation and protein stability regulate antigen presentation
• Kinase inhibitors may enhance antigen visibility for immunotherapy

## Graphical abstract

**Figure:**
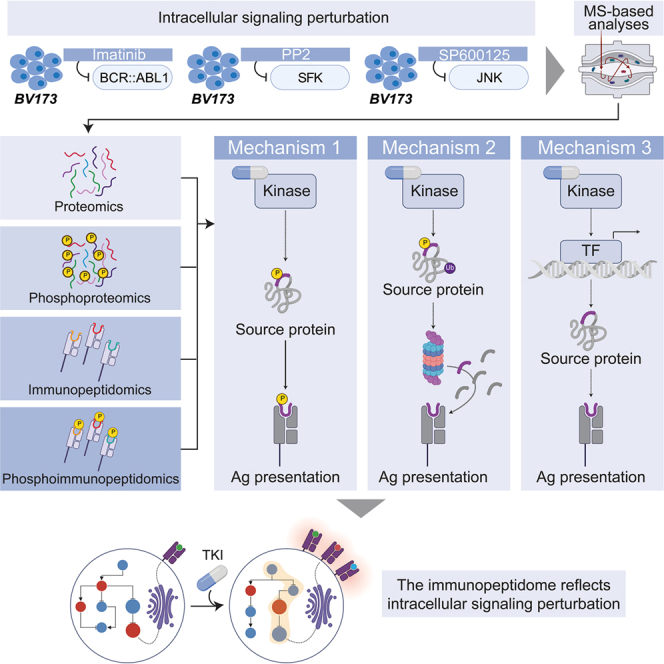

## Introduction

Chronic myeloid leukemia (CML) is defined by the t(9;22) chromosomal translocation, which results in the BCR-ABL1 fusion gene.^1^ The corresponding fusion protein exhibits constitutive tyrosine kinase activity and is effectively targeted by approved tyrosine kinase inhibitors (TKIs), dramatically improving the prognosis for CML patients.^2^ The current therapeutic objective in CML management is to achieve treatment-free remission (TFR) after discontinuation of BCR-ABL1 directed TKI treatment. TKI discontinuation is considered once patients sustain deep and durable molecular remission. However, only a subset of patients can stop TKI therapy without experiencing molecular relapse.^3^ As a result, long-term TKI therapy remains the standard of care for most patients, despite its potential for considerable side effects and the development of resistance.^4^ Multiple studies indicate that immune surveillance may play a crucial role in promoting TFR. Restoration of immune function—characterized by heightened activity of natural killer (NK) cells and T cells, along with decreased PD-1 expression on T cells^5^ —has been linked to the successful achievement of TFR. Therefore, enhancing CML-specific immune responses through T cell-based immunotherapy offers a potential route to increase the proportion of patients achieving durable TFR or even cure. Nonspecific immunotherapy strategies, including allogeneic stem cell transplantation and interferon-α (IFN-α) therapy, have been shown to induce long-lasting remissions following TKI discontinuation.^6^ More recently, immune checkpoint inhibitors—transformative in many solid tumors, are being explored in CML.^7^

More targeted immunotherapeutic approaches involve agents that induce immune responses against leukemia-specific antigens, such as peptide vaccines, T cell receptor (TCR)-mimic antibodies, and genetically engineered T cells.^8^^,^^9^ These strategies depend on the identification of tumor-associated, HLA-presented peptides that are recognized by cytotoxic T cells. While neoantigens resulting from tumor-specific mutations are key targets in solid tumors with high mutational burdens, their role in cancers with lower mutational loads, like CML, remains limited and uncertain. Advances in mass spectrometry (MS)-based immunopeptidomics have enabled large-scale identification of nonmutated, tumor-associated HLA peptides capable of eliciting peptide-specific T cell responses and serving as targets for T cell-based therapies.^10^ Successful immunotherapy relies on robust antigen presentation by cancer cells, which may be impaired by several factors, including aberrant signaling pathways. Pharmacological inhibition of specific kinases—such as CDK4, CDK6, and MEK—has been shown to enhance cancer cell immunogenicity by increasing tumor-associated antigen (TAA) presentation in other cancer types.^11^^,^^12^

However, a comprehensive characterization of the interaction between signaling pathways and TAA exposure is still lacking. Gaining deeper insight into this complex interplay is critical, as numerous FDA-approved kinase inhibitors are already in clinical use and, in principle, could be leveraged to tumor immunogenicity. To investigate this, we first used a computational network-based approach to identify kinases potentially involved in regulating antigen presentation. Subsequently, we applied high-sensitivity mass spectrometry (MS)-based proteomics quantitatively profile changes in class I HLA peptide repertoires following treatment with three different kinase inhibitors, including the standard-of-care agent, imatinib. This analysis revealed selective remodeling of the HLA-I immunopeptidome, with enhanced presentation of specific peptides, including previously undescribed TAAs, in response to defined kinase inhibitors.

To elucidate the signaling-dependent mechanisms underlying these alterations, we combined our immunopeptidomic data with mass spectrometry-based (phospho)immunopeptidome and (phospho)proteome profiling of kinase inhibitor-treated cells, together with a computational, network-based analysis. Through this integrative strategy, we uncovered three interlinked mechanisms connecting kinase activity to antigen display, involving phosphorylation of HLA-I-bound peptides, altered turnover of source proteins, and transcriptional regulation of antigen expression. Our findings underscore selective kinase inhibition as a potential approach to boost immunotherapy by enhancing the presentation of target antigens. Importantly, by characterizing the molecular mechanisms and signaling pathways that govern HLA-I antigen presentation, this work establishes a quantitative, network-based immunopeptidomics framework for systematically identifying and exploiting drug-induced HLA-I peptide display in next-generation immunotherapies.

## Results

### Identification of kinases controlling antigen presentation

To systematically identify key signaling molecules involved in the antigen presentation regulating TAA presentation, we applied the ProxPath network analysis, which estimates the functional proximity of signaling proteins to a target pathway using curated causal interactions from the SIGNOR resource.^13^ We focused on identifying druggable kinases relevant to CML pathobiology that are functionally connected to the antigen processing and presentation machinery (APPM) pathway. This analysis highlighted three kinases—BCR-ABL1, JNK, and Src-family kinase (SFK) signaling-as being linked to the APPM via causal paths associated with either upregulation or downregulation of antigen presentation (Figure S1A). Among them, SFK components showed the highest number of connections to the APPM (56 paths), suggesting a strong regulatory relationship.

### Immunopeptidomic profiling of kinase-inhibited CML cells

First, we investigated the impact of pharmacological inhibition of these three kinases on the antigen presentation. To this end, BV173 cells were treated for 72 h with imatinib (BCR-ABL inhibitor) 250 nM, SP600125 (JNK inhibitor) 10 μM and PP2 (Src-family kinase inhibitor) 25 μM. These concentrations were selected as they only modestly affected cell viability, thereby limiting potential confounding effects due to cytotoxicity (Figure S1B).

**Figure 1:**
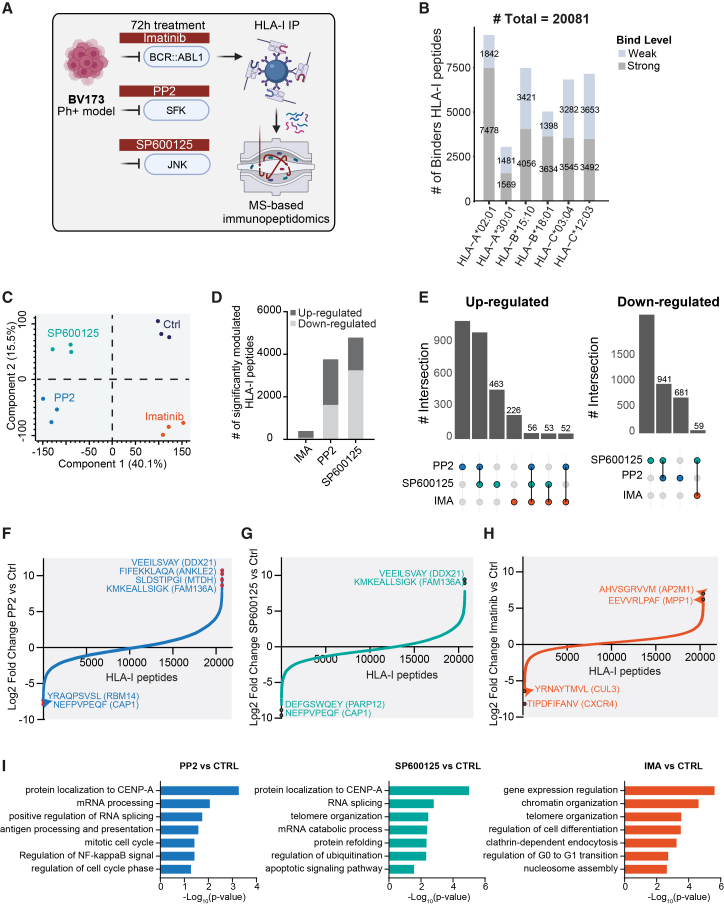
Quantitative analysis of HLA-I immunopeptidome profiling across kinase inhibitor treatments (A) Experimental workflow depicting the use of MS-based immunopeptidomics on the BV173 Ph+ chronic myeloid leukemia model treated for 72 h with 3 kinase inhibitors. (B) Number of peptides predicted by NetMHCpan 4.1 15 to bind distinct BV173 HLA-I alleles, separated by strong (gray) and weak (skyblue) binders. (C) Principal component analysis (PCA) of 21,078 HLA-I peptides quantified in BV173 cells treated with three different kinase inhibitor treatments (Imatinib, PP2, and SP600125) and control conditions. (D) Number of significantly up- and downregulated HLA-I peptides for each kinase inhibitor treatment relative to control. Significance was assessed using two-sample *t* tests (FDR <0.05). (E) Upset plots display the intersections of significantly upregulated (left) and downregulated (right) peptides among the different kinase inhibitor treatments. (F–H) Waterfall plots showing log_2_ fold changes in HLA-I peptide abundance upon treatment with PP2 (F), SP600125 (G), and imatinib (H) compared to control. Selected peptides with significant and strong fold changes and their source proteins are annotated. (I) Bar plots reporting the -log(*p* value) values for biological processes enriched by the source proteins of the HLA-I peptides significantly upregulated upon kinase inhibition.

We employed the recently developed IMBAS (immunopeptidomics by biotinylated antibodies and streptavidin) workflow^14^ in combination with high-sensitivity MS-based proteomics to quantify MHC-I-bound peptides in biological triplicates (Figure 1A). We used biotinylated W6/32 antibodies to immunoprecipitate antigenic peptides presented by MHC-I molecules, which were then captured on streptavidin beads. After washing, elution, and molecular weight filtration, samples were loaded onto Evotips for downstream MS analysis. This approach enabled the identification of 21,078 unique HLA-I peptides (Figure S1C) across the four experimental conditions. As expected, the peptides were highly enriched for nonameric sequences (Figure S1D). Most of the peptides (20,081 out of 21,078) were predicted to be strong or weak binders of the allelic profile of BV173 cells, by using NetMHCpan 4.1^15^ (Figure 1B and Table S1).

To assess how different kinase inhibitors influence the immunopeptidome, we analyzed the global changes in HLA-I-bound peptides following targeted treatments. First, to determine whether the three inhibitors could be classified in an unsupervised manner based on their impact on HLA-I peptide presentation, we applied principal-component analysis (PCA) to the immunopeptidomic dataset. This showed distinct and reproducible separation of the treatment groups, further supported by unsupervised hierarchical clustering and Pearson correlation analysis (Figures 1C, S1E, and S1F).

To identify HLA-I peptides significantly modulated by kinase inhibition, we performed a stringent two-sample *t* test analysis (Table S2). Treatment with SP600125 and PP2 affected more than 4,000 HLA-I peptides, whereas imatinib produced a milder response, altering the presentation of approximately 200 peptides (Figure 1D). Both induced substantial up- and downregulation of HLA-I peptides, with a considerable overlap in the modulated sets (Figure 1E). Notably, PP2 and SP600125 inhibition induced the strongest alterations in the abundance of surface-presented HLA-I peptides, with some antigens showing up to 1000-fold changes in expression (Figures 1F and 1G). Imatinib also reshaped the immunopeptidome, with some HLA-I peptides being up- or downregulated as much as 64-fold (Figure 1H). Importantly, the changes induced by all three inhibitors were observed across all major HLA-I subtypes (HLA-A, -B, and -C), indicating that their effects are not allele-specific (Figure S1G). Sequence motif analysis of significantly modulated peptides by SP600125, PP2, and imatinib revealed strong conservation with known binding motifs of the BV173 HLA alleles, particularly at anchor positions (Figures S2A–S2C), supporting the biological specificity of these alterations. Gene ontology (GO) enrichment analysis on the source proteins of the HLA-I peptides significantly upregulated by the three kinase inhibitors identified biological processes related to cell cycle control, chromatin organization, cell division, and antigen presentation, consistent with the expected biological response to imatinib, SP600125, and PP2 (Figure 1I).

### Multi-omics elucidates kinase-dependent mechanisms shaping HLA-I exposure

**Figure 2:**
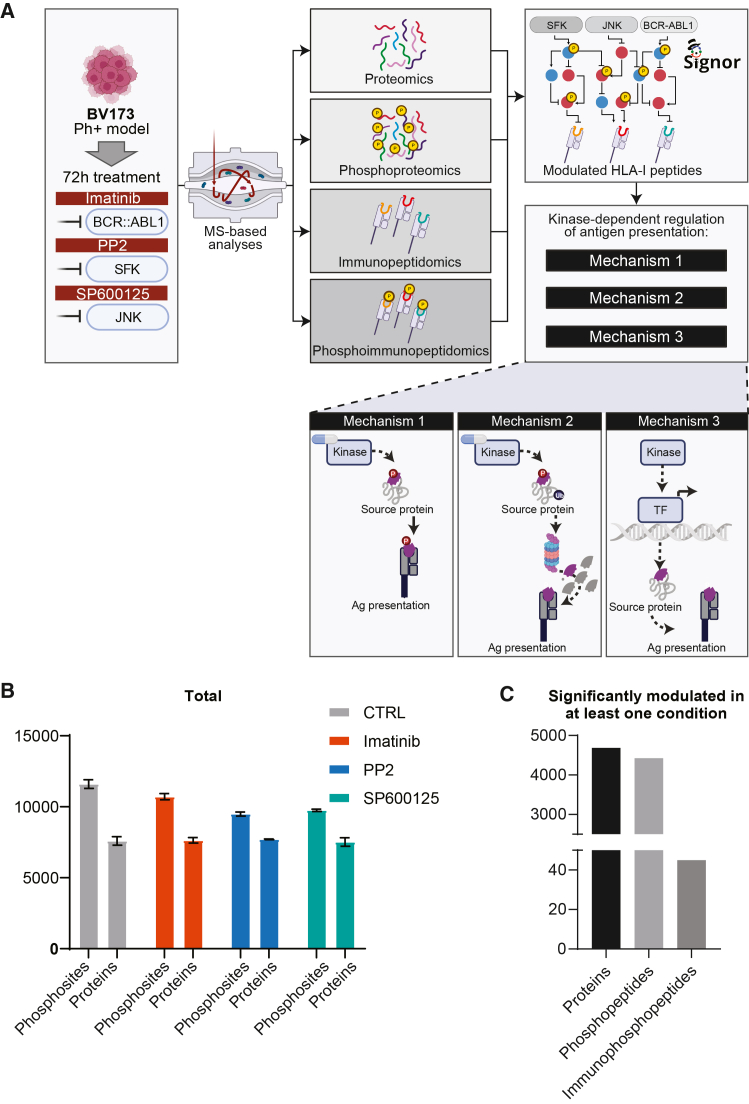
Application of a multi-step strategy to investigate treatment-dependent HLA-I modulation (A) Experimental workflow depicting the use of four different MS-based layers to ultimately identify the molecular mechanisms through which kinase inhibition can lead to a modulation of HLA-I expression. BV173 cells were treated with Imatinib, PP2, and SP600125 compounds for 72 h. Next, we performed for each condition, proteomics, phosphoproteomics, immunopeptidomics, and phospho-immunopeptidomics analyses. By integrating these four datasets, we identified three different mechanisms of antigen presentation modulations, (1) phosphorylation-dependent modulation of HLA-I peptides, (2) regulation of source protein stability, and (3) transcriptional regulation of source proteins. (B) Total number of proteins and phosphopeptides identified across treatments and control. (C) Number of significantly modulated proteins, phosphopeptides, and immunophosphopeptides in at least one condition compared to control. Data are presented as mean ± SEM.

Our findings show that inhibition of selected kinases leads to strong and selective up- or downregulation of hundreds of HLA-I peptides. To determine whether this effect is due to direct modulation of the APPM, we assessed MHC-I surface expression in BV173 cells after 72 h of treatment with PP2, SP600125, and imatinib using flow cytometry. HLA-I expression remained unchanged across all treatments, suggesting that SFK, JNK, and BCR-ABL1 regulate antigen exposure through indirect or post-translational mechanisms (Figure S2D). Because alterations in signaling, phosphorylation, and protein abundance are ultimately integrated into the repertoire of HLA-I-presented peptides, the immunopeptidome serves as a molecular readout of the intracellular state. To dissect the mechanisms underlying kinase-dependent modulation of HLA-I immunopeptidome presentation, we developed a network-based, multiomics strategy, combining our multi-omic data with the human causal network (Figure 2A). In addition to immunopeptidomic profiling, we analyzed three regulatory layers in BV173 cells treated with kinase inhibitors: phospho-immunopeptidomics, global phosphoproteomics, and total proteomics.

First, we re-analyzed the immunopeptidomic dataset to specifically identify phosphorylated HLA-I peptides (phospho-immunopeptidomic dataset). Next, we employed high sensitivity MS-based proteomics to quantify changes in the global proteome and phosphoproteome of BV173 cells after 72 h of treatment with PP2, SP600125, and imatinib. Using this strategy, we quantified about 60 phosphorylated HLA-I peptides (Table S3), more than 7700 proteins (Table S4 and Figure 2B) and 17,000 phosphosites (Table S5 and Figure 2B). Principal component analyses of the proteomics and phosphoproteomics datasets demonstrated high reproducibility and clear separation among treatments (Figures S3A and S3B). Statistical analyses (FDR <0.05) revealed that 45 phospho-HLA-I peptides (Table S3), 4,400 phosphosites, and 4,700 proteins were significantly modulated by at least one kinase inhibitor (Figures 2C, S3C, and S3D). We then integrated these multi-omic datasets with the human naive causal network to uncover the mechanisms by which kinase inhibition influences HLA-I peptide presentation. We found that BCR-ABL1, JNK, and SFK signaling reshape the CML immunopeptidome through three interrelated regulatory modes: (1) direct modulation of peptide phosphorylation, (2) stability-dependent control of source proteins, and (2) transcription factor (TF)-mediated regulation of peptide abundance. This approach allowed us to systematically try to map each modulated HLA-I peptide to one of these regulatory modes.

### Kinase-dependent HLA-I phosphorylation is associated with antigen exposure

**Figure 3:**
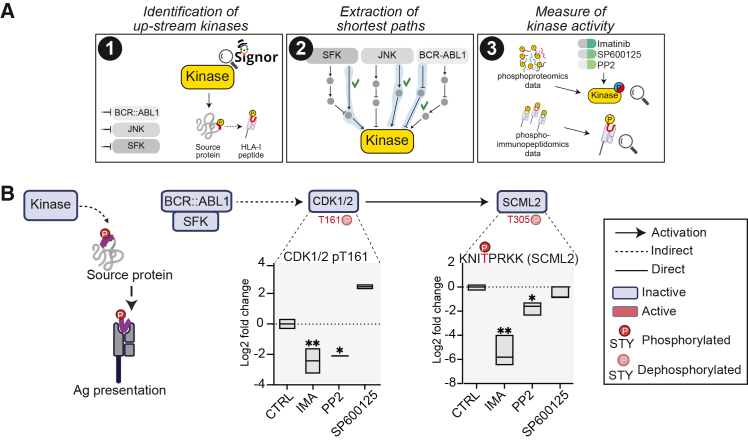
Kinase-dependent HLA-I phosphorylation is associated with antigen exposure (A) Illustration of the multi-step strategy we applied to identify HLA-I peptides modulated via direct phosphorylation. (B) Representative example illustrating how BCR-ABL and SFK kinases may influence HLA-I peptide phosphorylation. Boxplots show the log2 phospho-intensity of the activating site T161 on CDK1/2 (from phosphoproteomic data) and the log2 phospho-intensity of the SCML2-derived HLA-I peptide (from phospho-immunopeptidomic data). Statistical analysis was performed with Student’s *t* test analysis (∗*p* < 0.05, ∗∗*p* < 0.01, ∗∗∗*p* < 0.001).

Phosphorylation of HLA-I peptides has been correlated with enhanced affinity for HLA-I molecules.^16^ To assess the extent to which kinase inhibitors may reshape the immunopeptidome by influencing the phosphorylation of HLA-I peptides, we focused on the 45 significantly modulated phospho-HLA-I peptides and used the SIGNOR database to identify those that (1) contained a phosphosite with a known upstream kinase and (2) whose upstream kinase is itself directly or indirectly regulated by the inhibitor treatment (Figure 3A). When possible, we used our phosphoproteomic dataset to assess the activity of the upstream kinases upon drug treatment (Table S5). Although limited in number, direct phosphorylation-dependent regulation of HLA-I peptides was evident. Although our immunophosphoproteomic analysis has limited coverage and did not reveal phosphorylated peptides significantly affected by SFK inhibition, we observed that inhibition of JNK and BCR-ABL was associated with reduced presentation of the phosphorylated SCML2-derived HLA-I peptide KNITPRKK (Figure 3B).Due to the limited coverage inherent to our immunophosphoproteomic analysis, we didn’t find phosphorylated peptides significantly modulated by SFK inhibition. Network analysis further indicated that both imatinib and PP2 treatments were associated with decreased CDK1/2 activity, as indicated by decreased phosphorylation at the T161 activation site. This reduction coincided with decreased phosphorylation and surface presentation of the SCML2-derived HLA-I peptide (KNITPRKK), which contains the CDK1/2 substrate residue T305. Collectively, these observations suggest that kinase inhibition may be associated with changes in HLA-I peptide presentation through phosphorylation-linked processes, whereby drug-induced modulation of upstream kinases coincides with altered substrate phosphorylation and changes in the repertoire of peptides displayed at the cell surface.

### Kinase-associated modulation of source-protein stability is linked to HLA-I exposure

**Figure 4:**
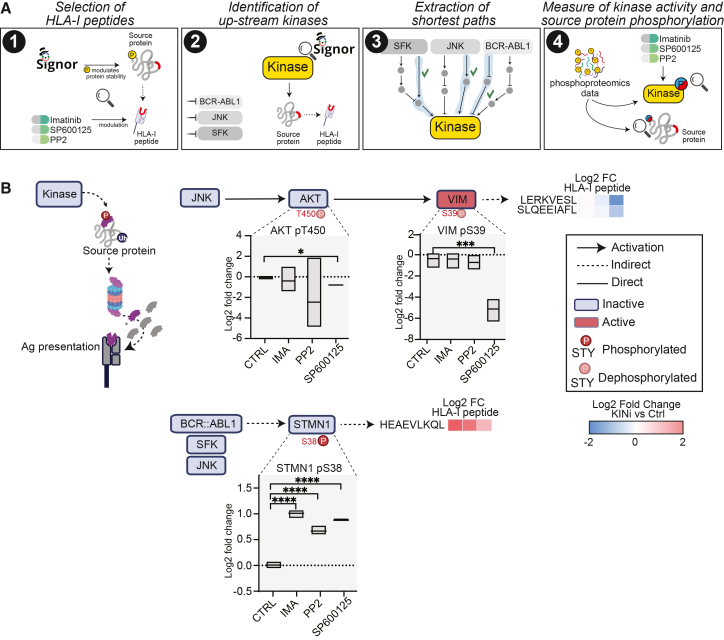
Kinase-associated modulation of source-protein stability is linked to HLA-I exposure (A) Illustration of the multi-step strategy we applied to identify HLA-I peptides modulated via regulation of source protein stability. (B) Proposed model depicting how kinase inhibition may regulate VIM and STMN1 stability via phosphorylation of key regulatory residues. Boxplots display phosphorylation levels of AKT (pS450), VIM (pS39), and STMN1 (S38) following inhibitor treatment (phosphoproteomic data). The heatmap summarizes the log2 fold changes in abundance of VIM and STMN1-derived peptides after kinase inhibition. Statistical analysis was performed with Student’s *t* test analysis (∗*p* < 0.05, ∗∗*p* < 0.01, ∗∗∗*p* < 0.001).

Phosphorylation of key residues can influence the proteasome-mediated degradation of proteins and consequently the availability of HLA-I peptides. We selected HLA-I peptides that were significantly modulated by at least one kinase inhibitor and derived from source proteins containing phosphosites known to regulate stability, as annotated in the SIGNOR database (Table S2) (Figure 4A). We further prioritized peptides whose upstream kinases were modulated by the inhibitors. When possible, we used our phosphoproteomic dataset to assess the activity of the upstream kinases and the phosphorylation level of the antigen source proteins upon kinase inhibition. This phosphorylation-dependent modulation of protein stability emerged as a predominant regulatory route, involving about 20 HLA-I peptides (Table S6). Notably, JNK inhibition was associated with a marked decrease in the presentation of two Vimentin (VIM)-derived HLA-I peptides. Our network-based analysis suggested a possible pathway linking JNK inhibition to the modulation of these VIM-derived peptides: JNK inhibition coincided with reduced AKT activation, as indicated by decreased phosphorylation at its activation site T450 in our phosphoproteomic dataset. Reduced AKT activity was associated with lower phosphorylation of VIM at S39 (Table S5), a modification previously linked to VIM stability. Consistent with this, these changes coincided with increased VIM stability and reduced presentation of VIM-derived HLA-I peptides (Figure 4B). Together, these results provide correlative evidence that kinase-dependent phosphorylation may influence HLA-I peptide presentation by modulating source protein turnover, ultimately influencing the repertoire of peptides displayed on the cell surface.

### Kinase-associated transcriptional changes coincide with altered HLA-I peptide exposure

**Figure 5:**
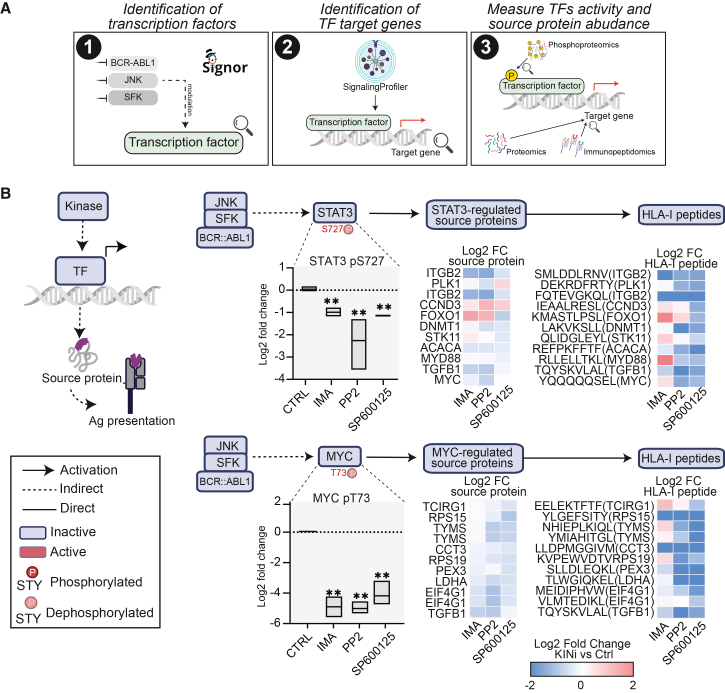
Kinase-associated transcriptional changes coincide with altered HLA-I peptide exposure (A) Illustration of the multi-step strategy we applied to identify HLA-I peptides modulated via transcriptional regulation of source protein. (B) Proposed model illustrating how kinase inhibitors may modulate transcription factors that regulate the expression of antigen source proteins. Boxplots present phosphorylation levels of STAT3 (pS727) and MYC (pT73) in response to inhibitor treatment (phosphoproteomic data). Heatmaps illustrate log2 fold changes in the abundance of STAT3-and MYC-regulated source proteins (proteomic data) and their corresponding HLA-I peptides (immunopeptidomic data). Statistical analysis was performed with Student’s *t* test analysis (∗*p* < 0.05, ∗∗*p* < 0.01, ∗∗∗*p* < 0.001).

Phosphorylation can also affect TF activity, thereby influencing gene expression and, consequently, antigen presentation. We identified HLA-I peptides derived from source proteins transcriptionally regulated by transcription factors whose activity is modulated by targeted kinases (Figure 5A). When available, we used our phosphoproteomic dataset to assess phosphorylation changes of transcription factors, while employing our proteomic and immunopeptidomic datasets we monitored the abundance of the downstream target source proteins and HLA-peptides, respectively. Transcriptional regulation emerged as a major layer of control, with 16 TFs regulating 170 source proteins and 222 associated HLA-I peptides (Table S6). For instance, our network-based strategy identified STAT3 and MYC as central nodes, collectively modulating ∼20 source proteins and their corresponding HLA-I peptides. Analysis of our (phospho)proteomic datasets showed that inhibition of BCR-ABL1, JNK, and SFK signaling coincided with reduced phosphorylation of STAT3 at S727 and MYC at T73, sites associated with their transcriptional activity. These phosphorylation changes were accompanied by decreased abundance of several STAT3-and MYC-associated source proteins and reduced presentation of their derived HLA-I peptides (Figure 5B). These findings suggest that kinase-associated phosphorylation of transcription factors may be linked to changes in HLA-I peptide presentation through transcriptional effects on antigen source proteins.

### SFK, BCR-ABL1, and JNK signaling are linked to altered presentation of tumor-associated antigens

**Figure 6:**
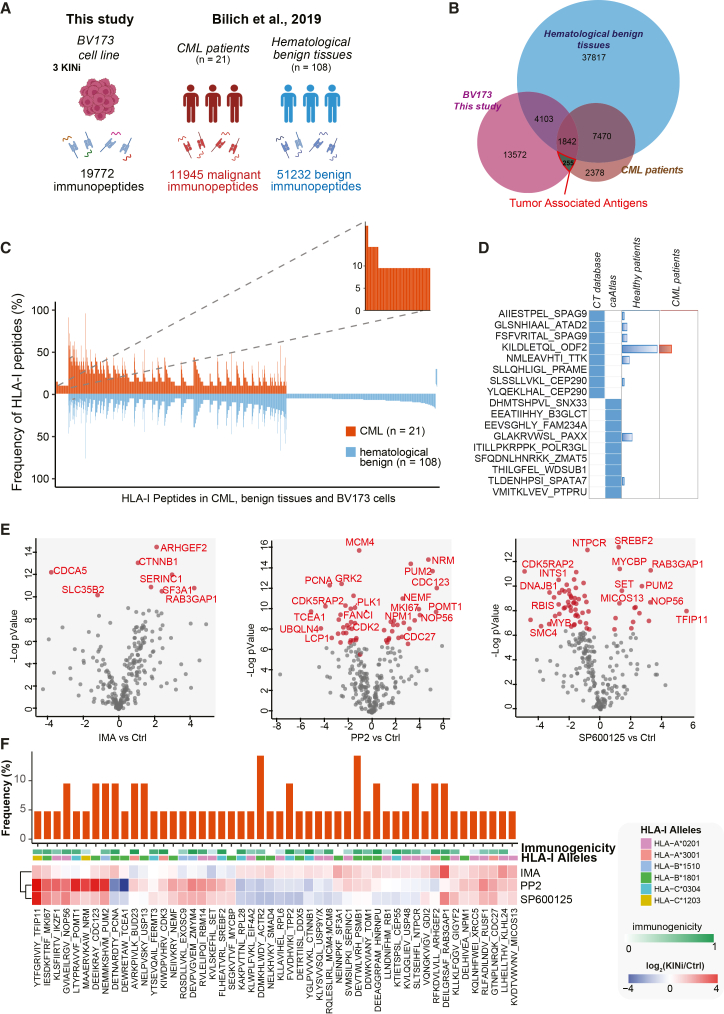
Identification and functional characterization of tumor associated antigens (A) Cartoon reporting the comparison between this study’s dataset with the Bilich et al.^10^ immunopeptidome dataset. (B) Venn Diagram reporting the overlaps in HLA-I peptide identification across BV173 binders, benign hematological tissues, and CML patient samples from Bilich et al.,^10^ study. (C) Frequency (*y* axis) distribution of BV173 HLA-I peptides (*x* axis) in CML patients and benign tissues.^17^ To allow for better readability, HLA peptides identified on >5% of the samples within the respective cohort are not depicted. The box on the left and its magnification highlight the subset of CML-associated antigens in BV173 cell lines showing CML-exclusive high frequent presentation. (D) Heatmap showing the cancer testis antigens, annotated in two databases^18^^,^^19^ and identified in our study. Bar plots show the number of CML or healthy (hematological tissues) displaying the selected antigen. (E) Volcano plots showing how kinase inhibition impacts the exposure of novel TAAs. (F) Heatmap showing the log2 fold-change of 22 tumor associated antigens significantly modulated in presentation by at least one kinase inhibitor and identified exclusively in CML patients. For each peptide, frequency in Bilich et al.,^10^ CML patients (barplot), the DeepImmuno predicted immunogenicity (green color scale) and the HLA allele in BV173 cells with strongest affinity are reported.

Our data show that inhibition of SFK and JNK signaling and, to a lesser extent BCR-ABL1, significantly reshapes the immunopeptidome of CML cells. We next asked whether kinase inhibition also impacts the abundance of well characterized tumor associated antigens (TAAs). To address this question, we compared our cell line-derived immunopeptidomic dataset with two independent reference cohorts: the immunopeptidome of a cohort of 21 primary CML samples and a comprehensive immunopeptidome of benign hematologic tissues^10^ (Figure 6A). This analysis revealed that approximately 10% of the HLA-I peptides identified in our study were also detected in primary CML samples but were entirely undetected in atlas of benign hematologic tissues (Figure 6B). We classified these 255 peptides as TAAs, collectively presented in about 10% of CML patients, highlighting their potential as immunotherapy targets (Figure 6C). We also assessed whether our dataset included known cancer-testis antigens (CTAs).^18^^,^^19^ CTAs are normally restricted to germ line cells (testis and placenta) but are aberrantly expressed in many cancers, making them promising immunotherapy targets.^18^^,^^19^^,^^20^ Our analysis identified 17 distinct HLA class I peptides derived from 15 CTAs. Notably, 7 out 17 antigens were also detected in benign hematologic samples and only one was identified in both CML and healthy primary samples (Figure 6D). Consistent with this, large-scale immunopeptidome profiling of 21 CML patients reported no frequent tumor-exclusive presentation of CTAs.^10^ These findings highlight the potential importance of identifying perturbations associated with enhanced presentation of the 255 CML-exclusive, non-mutated TAAs, which represent a potentially exploitable antigenic repertoire. We therefore examined how imatinib, PP2, and SP600125 were associated with changes in the abundance of these peptides. Quantitative immunopeptidomic profiling indicated that imatinib exerted relatively modest effects, whereas PP2 and SP600125 were associated with substantial changes in the exposure of 52 and 65 TAAs, respectively, including both up- and downregulation ranging from 4-fold to 64-fold (Figure 6E and Table S7). Remarkably, approximately 50 TAAs predicted to have high immunogenic potential were significantly upregulated in association with treatment with at least one kinase inhibitor (Figure 6F).

## Discussion

In this study, we provide a comprehensive, multi-omic characterization of the signaling-dependent mechanisms that regulate antigen presentation in CML cells. By integrating (phospho)-immunopeptidomics, global phosphoproteomics, proteomics, and a network-based computational framework, we demonstrate that inhibition of BCR-ABL1, JNK, and Src-family kinase (SFK) signaling profoundly reshapes the immunopeptidome of CML cells. In particular, pharmacological inhibition of these pathways not only alters the global abundance of HLA class I peptides but also selectively enhances the presentation of tumor-associated antigens (TAAs) with high predicted immunogenicity. These findings reveal novel opportunities for exploiting kinase inhibitors as adjuvants to immunotherapy in CML.

Consistent with this, inhibition of MEK and CDK4/6 has previously been shown to sensitize tumors to immunotherapy by upregulating MHC class I molecules, enhancing immune cell infiltration, activating T cells, and facilitating antigen recognition.^11^^,^^12^ While these studies described a general increase in antigen presentation upon kinase inhibition, our findings uncover a selective and fine-tuned mode of antigen modulation, indicating a higher level of specificity in the rewiring of the immunopeptidome.

A key conceptual advance of our work is the identification of three complementary mechanisms by which kinase inhibition modulates antigen exposure. First, direct phosphorylation of HLA-I peptides, although relatively rare, can be dynamically regulated by kinase signaling. Second, phosphorylation-dependent modulation of protein stability represents a more frequent mechanism, exemplified by AKT-mediated regulation of vimentin-derived peptides. Third, kinase inhibition alters TF activity leading to broad transcriptional changes in antigen source proteins and reshaping the immunopeptidome landscape.

Our analysis uncovered a unique repertoire of 255 non-mutated tumor-associated antigens (TAAs) that were consistently detected in primary CML samples but absent from benign hematologic tissues. This set of TAAs represents a valuable resource in the design of targeted immunotherapeutic strategies. Notably, classical cancer-testis antigens (CTAs) identified in our dataset, were also frequently detected in healthy hematopoietic samples. Such lack of CML exclusivity is consistent with previous large-scale immunopeptidomic studies, suggesting their limited clinical utility. In contrast, the 255 CML-exclusive peptides may offer more selective and safe targets for T cell-based therapies.

Together, these findings illustrate how perturbation of kinase activity can extend its effects across multiple molecular layers, ultimately reshaping the HLA-I-presented antigenic landscape. An important translational implication of our work is that targeted kinase inhibition can be leveraged to enhance the presentation of immunogenic antigens. While imatinib had only modest effects on TAA presentation, SFK and JNK inhibition caused extensive remodeling of the immunopeptidome, altering the abundance of more than 100 TAAs. Importantly, a subset of approximately 50 TAAs was predicted to be highly immunogenic and also upregulated by kinase inhibitor treatment. Overall, these results suggest that rational combinations of kinase inhibitors with T cell-based immunotherapy could enhance antigen visibility and immune recognition, thereby increasing the likelihood of durable TFR in CML.

While these results are promising, several limitations warrant consideration. First, the use of a single CML cell line may not fully capture the biological and immunopeptidomic heterogeneity of CML across different disease stages and lineages. Consequently, kinase inhibitor-induced immunopeptidome remodeling observed in this model may differ in other CML settings, and future validation in additional CML models and/or complementary in silico analyses will be required to assess the broader applicability of these findings. Second, despite pronounced remodeling of the immunopeptidome, we found that HLA class I surface expression was unchanged upon kinases inhibition but we did not directly assess individual components of the antigen processing and peptide-loading machinery in this study. Indeed, kinase inhibition may alter peptide repertoire composition through changes in antigen processing efficiency, proteasomal activity, peptide availability, or TAP- and chaperone-mediated peptide loading. Third, while our data support an association between signaling rewiring and changes in HLA peptide presentation, we acknowledge that the proposed mechanisms are inferred from correlative network-level analyses. Future works including targeted pathway inhibition or genetic perturbation of key intermediates, together with direct measurements of substrate phosphorylation and protein half-life, will be necessary to rigorously test causality and to validate the contribution of protein stability to the observed immunopeptidomic shifts.

Additionally, kinase inhibitors often exhibit off-target effects, and their pleiotropic nature should be carefully considered in the interpretation of signaling dependencies and in the design of future therapeutic combinations. Future studies incorporating complementary genetic approaches or the use of more selective inhibitors will be essential to further delineate pathway specificity.

Although the 255 TAAs identified in this study were also presented in primary CML tissues,^10^ as BV173 is derived from lymphoid blast crisis, the TAA signatures we identified may not fully reflect those present in chronic-phase CML primary cells or in other genetic subtypes. Therefore, further validation across a wider range of models will be necessary to distinguish broadly conserved targets from those that are context-dependent. Moreover, further validation is needed to confirm their functional immunogenicity and capacity elicit effective anti-tumor T cell responses. In this context, immunopeptides that are upregulated upon inhibitor treatment represent the most relevant candidates for future translational applications, as their increased presentation is expected to enhance tumor immune recognition. These peptides could represent a starting point for future experimental validation, including *in vitro* T cell activation assays and *in vivo* models, and, if confirmed, may ultimately support the development of antigen-specific immunotherapeutic or vaccine-based strategies in CML.

In conclusion, our findings indicate pharmacological inhibition of signaling pathways as a strategy not only to kill cancer cells but also to enhance their immunogenicity by increasing tumor antigen visibility. To our knowledge, this is the first study that demonstrates that inhibition of specific kinases affects the presentation of specific HLA-I peptides, including previously undescribed TAAs, and to provide a mechanistic basis for this kinase-dependent modulation. By delineating signaling-driven mechanisms and uncovering a set of CML-exclusive TAAs, we establish a framework for leveraging kinase inhibitors as tools to reshape the HLA-I immunopeptidome and enhance the effectiveness of immunotherapy. Future work should focus on validating the immunogenicity of these peptides and test rational combinations of kinase inhibitors with immunotherapy in preclinical and clinical settings, with the ultimate goal of expanding the proportion of patients who achieve durable TFR.

### Limitations of the study

This study has several limitations. First, our analyses were primarily performed in a single CML cell line model, which may not fully capture the biological and immunopeptidomic heterogeneity of CML across different disease stages, cellular lineages, and patient-specific genetic backgrounds. As BV173 cells are derived from lymphoid blast crisis, the antigen repertoire and signaling dependencies identified here may differ from those present in chronic-phase CML or other disease contexts. Therefore, further validation in additional CML models and primary patient samples will be necessary to assess the generalizability of these findings. Second, although kinase inhibition induced substantial remodeling of the immunopeptidome, we did not directly investigate individual components of the antigen processing and peptide-loading machinery. Changes in peptide presentation may result from alterations in proteasomal processing, peptide transport, chaperone-mediated loading, or protein degradation rates, which were not directly measured in this study. Third, the proposed mechanisms linking kinase signaling, protein phosphorylation, protein stability, and antigen presentation are largely based on integrative and network-level correlative analyses and therefore do not establish direct causality. Future studies using targeted genetic perturbations, selective inhibitors, and direct measurements of protein phosphorylation and stability will be necessary to validate these mechanisms. Additionally, kinase inhibitors can exhibit off-target effects, and their pleiotropic activity should be considered when interpreting signaling dependencies and immunopeptidome changes. Finally, although we identified a set of CML-associated tumor-associated antigens and predicted their immunogenicity, their ability to elicit functional anti-tumor T cell responses remains to be experimentally validated. Future work should include T cell activation assays and validation in primary samples and *in vivo* models to determine their translational potential.

## Resource availability

### Lead contact

Requests for further information and resources should be directed to and will be fulfilled by the lead contact, Francesca Sacco (francesca.sacco@uniroma2.it).

### Materials availability

This study did not generate new unique reagents.

### Data and code availability

• Mass spectrometry data have been deposited to the ProteomeXchange Consortium via the PRIDE partner repository with the dataset identifier PXD076608 and are publicly available as of the date of publication. Accession codes are also listed in the key resources table.
• This paper does not report original code.
• Any other data supporting the findings of this study are available from the corresponding authors upon request.

## Quantification and statistical analysis

All experiments were performed in biological triplicates (*n* = 3 independent cell culture samples per condition). Peptide and protein intensities were log_2_ transformed prior to analysis, and missing values in the immunopeptidomic dataset were imputed using the MinProb method (q = 0.01) from the DEP R package (v. 1.28.0); only peptides detected in at least 2 out of 3 replicates in at least one condition were retained. Differentially modulated peptides and proteins were identified using two-sample Student’s t-tests, with significance defined as absolute log_2_ fold-change >0.5 and adjusted *p*-value <0.05 (Benjamini-Hochberg correction). For phospho-immunopeptidomic comparisons (Figures 3, 4, and 5), significance thresholds are indicated as ∗*p* < 0.05, ∗∗*p* < 0.01, ∗∗∗*p* < 0.001. Data are presented as mean ± SEM unless otherwise stated; flow cytometry data are reported as mean ± SD. Statistical analyses were performed in R, DIA-NN v. 2.0, and Spectronaut v. 20.

## Acknowledgments

This research was funded by the Italian Association for Cancer Research (AIRC) with a grant to L.P. (MFAG Grant no. 28858) and a grant to F.S. (Start-Up Grant no. 21815) and by MUR PRIN 2022 (no. E53D23004850006). G.M. is supported by MUR PRIN 2022 (no. E53D23004850006). L.P. and F.S. are supported by a joint PRIN 2022 PNRR grant (no. P2022JRETW), funded by the European Union – NextGenerationEU, and by a SEED Sapienza Grant. V.V. is supported by PON-MUR fellowship (no. DOT13IEP1U-1).

## Author contributions

Conceptualization, G.M., L.P., and F.S.; resources, M.W., G.M., and V.B.; software, V.V. and L.P.; methodology, V.V., M.W., G.M., V.B., L.P., and F.S.; formal analysis, V.V., M.W., G.M., and V.B.; investigation, V.V., M.W., G.M., and V.B., with the contribution of T.F., M.B., and D.M.; writing – original draft preparation, V.V., M.W., G.M., L.P., and F.S.; writing – review and editing, all; supervision, L.P. and F.S.; funding acquisition, F.S. All authors have read and agreed to the published version of the manuscript.

## Declaration of interests

The authors declare no competing financial interests.

## STAR★Methods

### Key resources table

**Table:** 

REAGENT or RESOURCE	SOURCE	IDENTIFIER
**Antibodies**		

W6/32 antibody	*In Vivo* Biosciences	Custom made
Human HLA Class I APC-conjugated Antibody	R&D Systems	FAB7098A

**Deposited data**		

Mass Spectrometry data	PRIDE	PXD076608

**Experimental models: Cell lines**		

BV173	DSMZ	ACC 20

**Software and algorithms**		

DIANN v.2.0	GitHub	https://github.com/vdemichev/DiaNN
NetMHCpan 4.1	Technical University of Denmark;	https://services.healthtech.dtu.dk/services/NetMHCpan-4.1/
ProxPath	GitHub	https://github.com/saezlab/ProxPath
Perseus	Max Planck Institute of Biochemistry	https://maxquant.net/perseus/
CytExpert	Beckman Coulter	http://beckman.it/flow-cytometry/research-flow-cytometers/cytoflex/software

### Experimental model and study participant details

BV173 cell line were purchased from DSMZ (ACC 20) and cultured in DMEM medium (Thermo Scientific, 41965062) supplemented with 10% heat-inactivated fetal bovine serum (Aurogene, AU-S1810-500), and 100 U/ml penicillin and 100 mg/mL streptomycin (Gibco 15140122). Cells were routinely tested for mycoplasma contamination (Mycoplasma PCR Detection Kit, Abcam, ab289834). BV173 cells were seeded at 500.000 cells/mL and treated with the following inhibitors: 250 nM imatinib (Selleck Chemicals, S2475), 25 μM PP2 (Selleck Chemicals, S7008), or 10 μM SP600125 (Selleck Chemicals, S1460) for 72 h. Control cells were left untreated under identical culture conditions. Treatments were performed only on cultures with >90% viability, as assessed by trypan blue exclusion prior to drug exposure.

### Method details

#### ProxPath analysis

The ProxPath algorithm (https://github.com/saezlab/ProxPath) was used to calculate the number of paths connecting each SIGNOR protein (input node) to the members of the *MHC class I antigen presentation* pathway (https://signor.uniroma2.it/pathway_browser.php?beta=3.0&organism=&pathway_list=SIGNOR-MCIAP&x=14&y=10). Input nodes with high number of paths impacting onto the antigen processing and presentation machinery (APPM) pathway, short distance with respect to the average distance (*Z* score <0), and available inhibitors were selected for perturbation, namely SFK, BCR-ABL1 and JNK.

#### Proteomic sample preparation

Cell pellets were dissolved in 100 μL lysis buffer (10% AcN, 60 mM TEAB, 5 mM TCEP, 25 mM CAA) and incubated at 76°C for 20 min on an orbital shaker. Next, samples were sonicated in a Bioruptor (Diagenode) for 10 cycles with 50% duty cycle. 2 μg of each trypsin and Lys-C were added for protein digestion and incubated overnight at 37°C. The digestion was stopped by adding formic acid (FA) to a final concentration of 1%. To clear them from cellular debris, samples were centrifuged at 5000 xg for 3 min in a benchtop centrifuge (Eppendorf). 5 μL of the digest were saved for full proteome measurement. From the remaining digest, phosphorylated peptides were enriched on an AssayMAP Bravo Sample Prep Platform (Agilent), using the Phosphopeptide Enrichment v2.1 Protocol in the Protein Sample Prep Workbench v3.2.0 with standard settings. 5 μL Fe-NTA Cartridges (Agilent) were primed with 0.1% trifluoroacetic acid (TFA) in AcN. Samples were loaded on the cartridges, washed with 0.1% TFA in 80% AcN and finally eluted in 0.5 M ammonium dihydrogenphosphate solution. Both full proteome and phosphoproteome samples were loaded on Evotips (Evosep) according to the manufacturer’s protocol. In brief, Evotips were activated with isopropanol, primed two times with 50 μL of Buffer B (0.1% FA in AcN) and equilibrated two times with 50 μL of Buffer A (0.1% FA). Samples were loaded on activated tips, washed twice with Buffer A and stored at 4°C until measurement. All centrifugation steps were done at 700 xg for 1 min.

#### Immunopeptidomics enrichment

Immunopeptidomics enrichment was performed using the IMBAS-MS workflow.^14^ In brief, cell pellets were resuspended in 200 μL lysis buffer (50 mM Tris, pH8;150 mM NaCl, 0.5% NP-40; 60 mM Octyl-b-D-glucopyranosid; 1 x cOmplete Protease inhibitor) and incubated for 30 min on ice. Samples were centrifuged for 20 min at 4°C at 15.000 rcf and the supernatant is transferred to a fresh tube. Samples were incubated overnight with 10 μg W6/32 antibody (*In Vivo* Biosciences Custom made) at 4°C. Enrichment was performed using 20 μL magnetic streptavidin beads. Beads were washed in 3 steps (1: 10 mM Tris pH8, 150 mM NaCl; 2: 10 mM Tris pH8, 450 mM NaCl; 3: 10 mM Tris pH8) before elution in 200 mM Glycine at pH2. The eluent was centrifuged through a 10 kDa flat bottom tube filter (Merck) before loaded onto Evotips following the same procedure described above.

#### Mass spectrometry analysis

The proteome and phosphoproteome LC/MS measurements were carried out using an EvoSep One liquid chromatography system (Evosep) coupled to a trapped ion mobility spectrometry quadrupole time-of-flight mass spectrometer (timsTOF HT, Bruker Daltonik) via a nanoelectrospray ion source. Samples were analyzed using a 44 min gradient (30 SPD) eluting the peptides at 250 nL/min flow rate. We used a 15 cm × 75 μm column with 1.9 μm C18 beads (EvoSep) and a 10 μm ID zero dead volume electrospray emitter (Bruker Daltonik). Mobile phases A and B were 0.1% FA in water and 0.1% FA in AcN, respectively. For proteome measurements, 20 isolation windows were placed between an m/z range of 350–1200 and an ion mobility (IM) range between 1.3 and 0.7 V cm-2 at a 2.2 s cycle time. The collision energy was decreased as a function of the IM from 59 eV at 1/K0 = 1.6 to 20 eV at 1/K0 = 0.6. For phosphoproteome measurements the mass range was shifted to m/z 400 to 1400 and the ion mobility (IM) range was shifted to 1.45 and 0.75 V cm-2. The collision energy was adjusted toward 60 eV at 1/K0 = 1.5 to 54 eV at 1/K0 = 1.17 to 25 eV at 1/K0 = 0.85 and end at 20 eV at 1/K0 = 0.6. The immunopeptidome LC/MS measurements were carried out using an EvoSep One LC system coupled to a timsTOF Ultra 2. Samples were separated using a 31 min gradient (whisper 40) over an Aurora IonOpticks 15 cm column. For acquisition, the same methods were employed as previously published (ref IMBAS paper).

#### Proteome and phosphoproteome raw data processing

Full proteome raw files were analyzed with DIA-NN v. 2.0 (https://github.com/vdemichev/DiaNN) and phosphoproteome samples with Spectronaut version 20. MS/MS spectra were matched against the Homo sapiens UniProtKB FASTA database, with an FDR of <1% at protein-, peptide- and modification-level. Enzyme specificity was set to trypsin with the maximum number of missed cleavages set to 1. Cysteine carbamidomethylation was added as a fixed modification, variable modifications were set to N-terminal protein acetylation and oxidation of methionine as well as phosphorylation of serine, threonine tyrosine residue (STY) for the phosphoproteomic samples.

#### Immunopeptidome raw data processing

Immunopeptidome raw files were analyzed using DIA-NN v.2.0 against a predicted panHLA library.^14^ Results were filtered at a peptide FDR of 1% without applying a protein FDR. Phosphoimmunopeptidome results were obtained with Spectronaut version 20. Raw files were searched using the phosphor PTM workflow while setting the digestion type to unspecific and restricting the peptide length to minimum 7 and maximum 15. The peptide FDR was kept at 1% while the protein FDR filter was disabled. The PTM probability cut-off was set at 0.75.

#### Data preprocessing

Immunopeptides’ source proteins were annotated using the HLA Ligand Atlas dataset^17^ using immunorelated tissues (“Bone marrow”, “Lymph node”, “Myelon”, “Spleen”, “Thymus”) and the UNIPROT database. Intensities were log2 transformed, and only immunopeptides detected in 2 out of 3 replicates of at least one condition were retained. Missing values were imputed using the MinProb method (q = 0.01) from the DEP R package (v. 1.28.0) to simulate low-abundance signals.

#### MHC binding affinity and immunogenicity prediction

Binding affinities of immunopeptides to HLA-I alleles were predicted using the NetMHCpan 4.1 web application (https://services.healthtech.dtu.dk/services/NetMHCpan-4.1/),^15^ with the BV173 cell line HLA-I allele set (HLA-A02:01, HLA-A30:01, HLA-B15:10, HLA-B18:01, HLA-C03:04, and HLA-C12:03). Immunogenicity was assessed using the DeepImmuno web application (https://doi.org/10.1093/bib/bbab160), considering only peptides of length 9 or 10.

#### Identification of tumor-associated antigens

Tumor-associated antigens (TAAs) were defined as BV173 MHC-I-binding immunopeptides that were also detected in 21 CML patients from the Bilich et al., study^10^ but not present in the benign peptide set reported in the same study. This resulted in a list of 255 peptides. From these, we selected as putative TAAs 94 immunopeptides that were significantly overexpressed in response to at least one kinase inhibitor.

#### Mechanisms of regulations characterization

To characterize the mechanisms underlying the modulation of HLA-I peptide presentation, we integrated our multi-omic dataset with a literature-derived causal network.

Specifically, to investigate how kinase inhibition directly affects the exposure of phosphorylated HLA-I peptides (*Mechanism 1*), we applied the following strategy.

1. First, we queried the SIGNOR database to identify the upstream kinase of the phosphorylated HLA-I peptide source protein modulated upon kinase inhibition (Table S2).
2. Next, we extracted the shortest paths linking each upstream kinase to the inhibited kinases (BCR-ABL, LCK, JNK).
3. We then used our phosphoproteomic data (Table S5) to assess the activity of the upstream kinases upon drug treatment.

To identify HLA-I peptides modulated via phosphorylation-dependent changes in source protein stability (*Mechanism 2*), we used the following approach.

1. First, we selected only those HLA-I peptides significantly affected by kinase inhibition (Table S2) and mapped to source proteins whose phosphorylation has been reported to regulate protein stability in the SIGNOR database.
2. Next, we queried the SIGNOR database to identify the upstream kinase phosphorylating the source proteins.
3. We extracted the shortest paths linking each upstream kinase to the inhibited kinases (BCR-ABL, LCK, JNK), retaining only paths involving no more than two steps.
4. When the data were available, we used our phosphoproteomic data to assess the activity of the upstream kinases and the phosphorylation level of the source proteins upon kinase inhibition.

To identify HLA-I peptides modulated via phosphorylation-dependent modulation of transcription factors (*Mechanism 3*), we used the following strategy.

1. We used SIGNOR to identify transcription factors directly or indirectly regulated by the inhibited kinases.
2. We retrieved each transcription factor’s downstream target genes from the SignalingProfiler TF–target database (SIGNOR + Collectri).
3. When the data were available, phosphorylated transcription factors were compared with our phosphoproteomic data, while their downstream target genes were matched against the proteomic and immunopeptidomic datasets.
4. We retained HLA-I peptides showing consistent changes in expression aligned with both transcription factor activity and kinase inhibition.

#### Flow cytometry analysis of MHC class I expression

BV173 cells were plated at a density of 1 × 10^6^ cells/mL and treated with each inhibitor for 72h. After treatment, cells were washed with FACS buffer (1X PBS, 1 mM EDTA, 1% FBS, 0.1% NaN_3_) and incubated with anti-HLA Class I Alexa Fluor 647 conjugated antibody (R&D Systems, FAB7098A) using 1 μg antibody per 100 μL of cell suspension for 30 min at 4°C. Cells were washed 2X with FACS buffer, prior to analysis on CytoFLEX flow cytometer (Beckman Coulter Life Sciences). CytExpert software was used for data analysis. Cells were gated for live populations only. The percentage of HLA-I-positive cells was calculated and plotted for each sample as the average +/− SD.

## Supplemental information

Document S1. Figures S1–S3

Table S1. HLA-I immunopeptidomic dataset and MHC-I binding affinity predictions for BV173 cells across kinase inhibitor treatments

Table S2. Statistical analysis of the HLA-I immunopeptidome of BV173 cells treated with kinase inhibitors compared to control

Table S3. Phosphorylated HLA-I peptides identified by phospho-immunopeptidomics in BV173 cells

Table S4. Proteomic dataset of BV173 cells treated with kinase inhibitors

Table S5. Phosphoproteomic dataset of BV173 cells treated with kinase inhibitors

Table S6. Multi-omic data supporting the three proposed mechanisms of kinase-dependent immunopeptidome modulation

Table S7. Statistical analysis of tumor-associated antigens (TAAs) modulated by kinase inhibitor treatment in BV173 cells
