# Supplementary material for: Kinase inhibition rewires the HLA-I immunopeptidome in chronic myeloid leukemia
Source: iScience. 2026 Apr 30;29(6):115944. doi: 10.1016/j.isci.2026.115944 (PMC13196390; doi:10.1016/j.isci.2026.115944)
Supplement: Document S1. Figures S1–S3 [file mmc1.pdf]

iScience, Volume *29*

## Supplemental information

# Kinase inhibition rewires the HLA-I immunopeptidome in chronic myeloid leukemia

**Veronica Venafra, Maria Wahle, Giorgia Massacci, Valeria Bica, Patrizia Chiusolo, Dimitrios Mougiakakos, Martin Boettcher, Thomas Fischer, Livia Perfetto, Matthias Mann, and Francesca Sacco**

## Supplementary figures

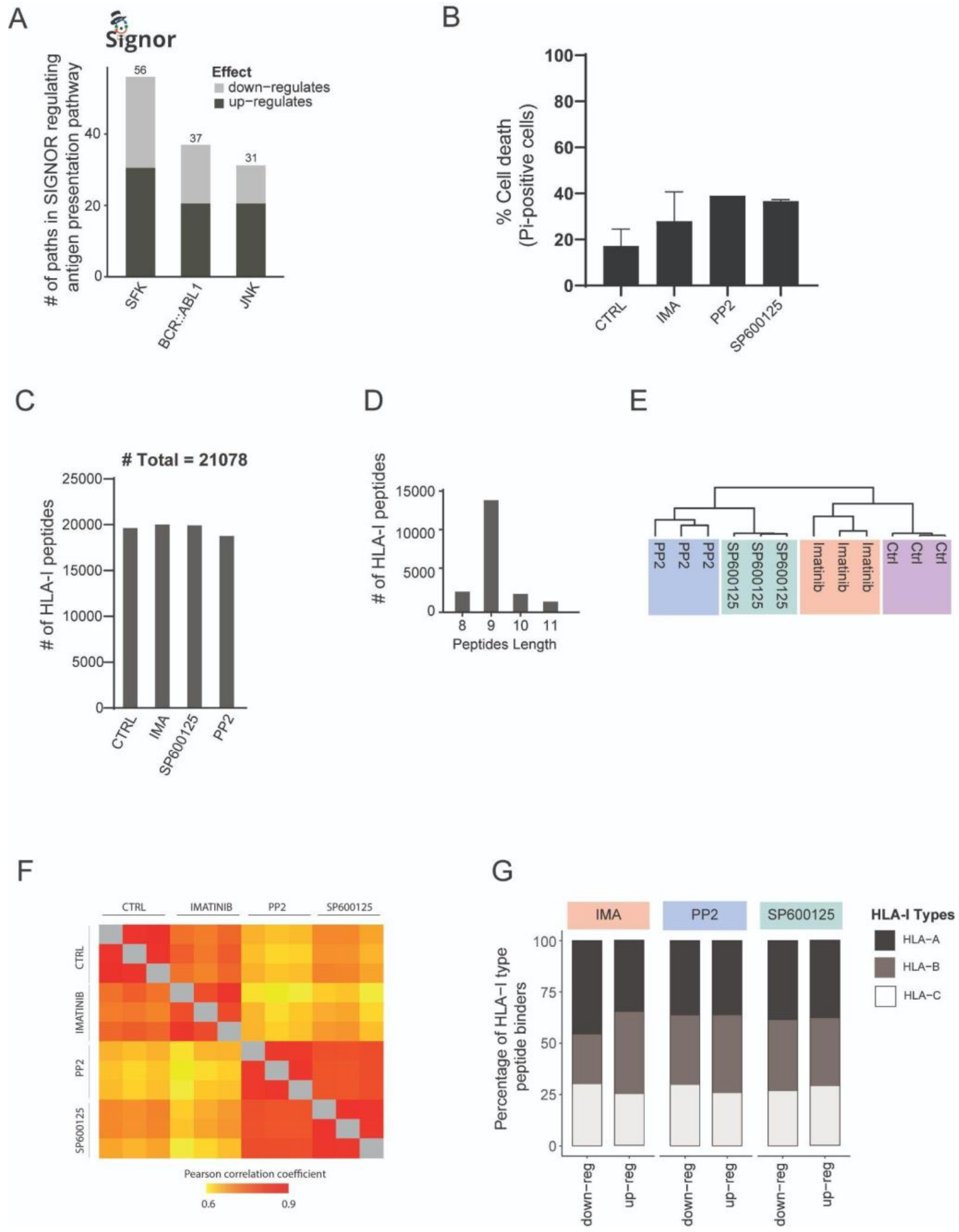


**Figure S1. A. MS-based immunopeptidomic analysis of BV173 cells upon Imatinib, PP2 and SP600125 treatment.** Barplot reporting the number of paths through which the closest kinases impact the antigen presentation pathway, as predicted by the ProxPath algorithm. **B**. Bar plot reporting the percentage of dead cells upon kinase inhibition with Imatinib 250nm, PP2 10μM and SP600125 25μM. The percentage of dead cells was calculated with Pi staining and flow cytometry analysis. **C.** Total number of HLA-I peptides identified across treatments and control. **D.** Length distribution of the identified HLA-I peptides. **E**. Unsupervised, hierarchical clustering (Pearson correlation distance) based on the abundance of 21,078 HLA-I peptides. **F**. Heatmap reporting the Pearson correlation coefficients of the immunopeptidomic analysis between the biological replicates. **G**. Stacked barplots showing the percentage of significantly up- and down-regulated peptides for HLA-A, HLA-B, and HLA-C alleles. modulated HLA-I binders across HLA-A, -B, and -C alleles among up- and down-regulated peptides in cells Imatinib, PP2, and SP600125 treatments.

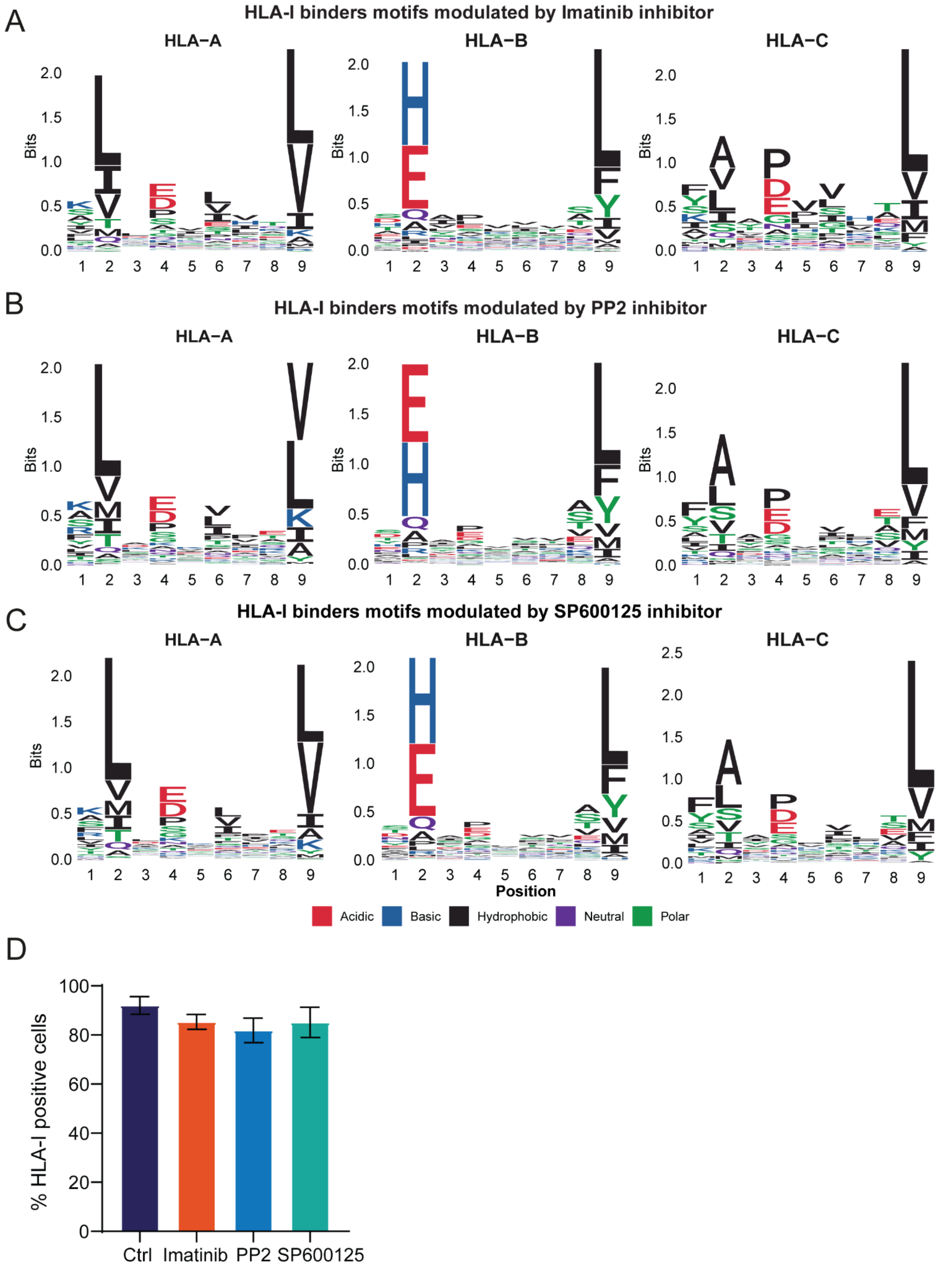


**Figure S2. HLA-I binding motifs analysis. A-C.** Sequence motifs of HLA-I peptides modulated by Imatinib (**A**), PP2 (**B**), and SP600125 (**C**) treatments, stratified by HLA-A, HLA-B, and HLA-C alleles. **D.** Flow cytometry analysis to assess HLA-I positive BV173 cells upon 48 hours treatment with imatinib, PP2, and SP600125.

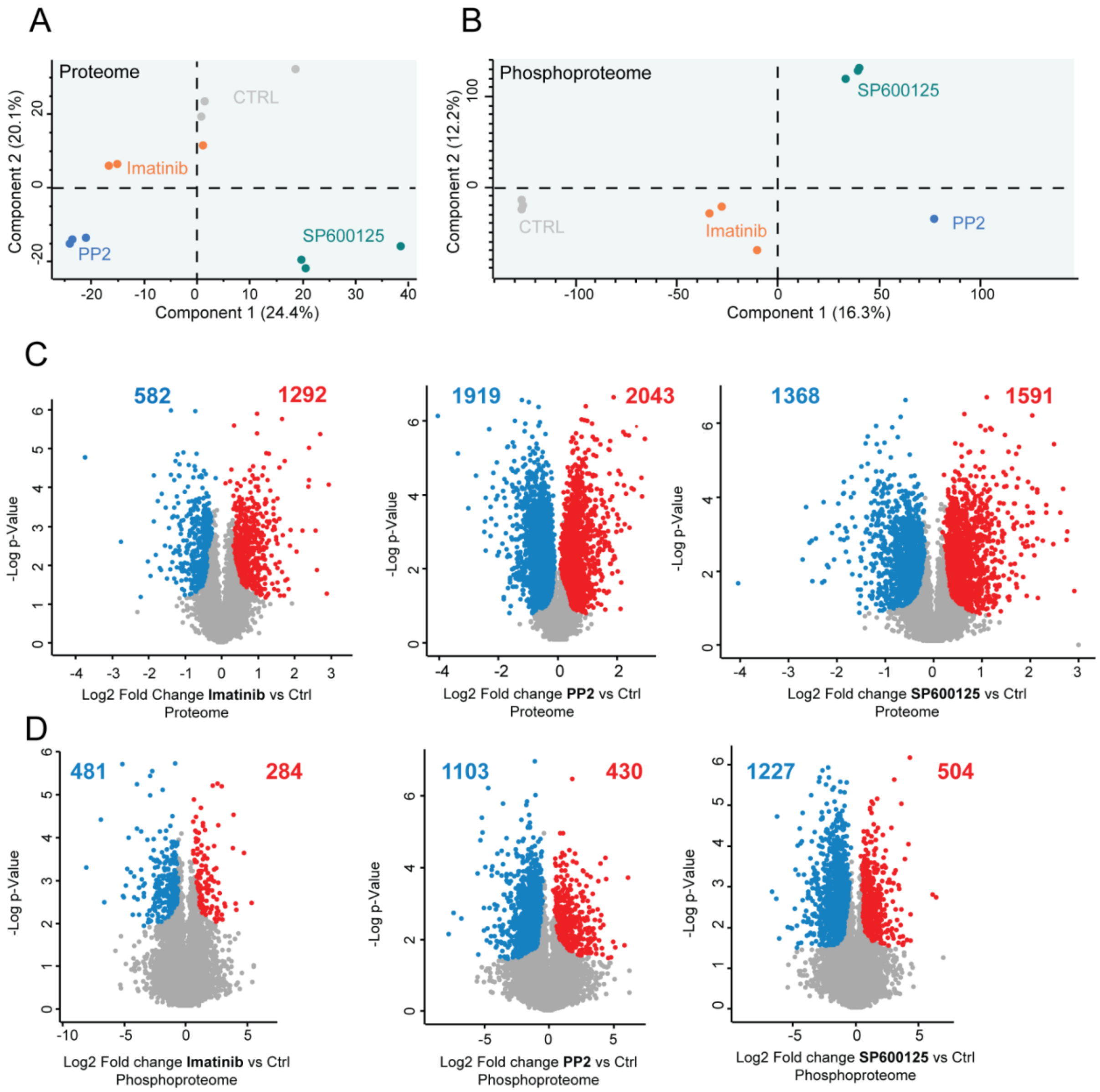


**Figure S3. MS-based (phospho)-proteomic analysis of BV173 cells upon Imatinib, PP2 and SP600125 treatment. A-B.** Principal component analysis (PCA) of proteins (**A**) and phosphopeptides (**B**) quantified in BV173 cells treated with three different kinase inhibitor treatments (Imatinib, PP2, SP600125) and control conditions. **C.** Volcano plots showing significantly up-regulated (red) and down-regulated (blue) proteins in imatinib, PP2, and SP600125 samples compared to control condition. Proteins that are not significantly modulated are represented in grey. how kinase inhibition impacts the exposure of novel TAAs. **D.** Volcano plots showing significantly up-regulated (red) and down-regulated (blue) proteins in imatinib, PP2, and SP600125 samples compared to control condition. Proteins that are not significantly modulated are represented in grey. how kinase inhibition impacts the exposure of novel TAAs.
